# Genetic Diversity of *Legionella pneumophila* Isolates from Artificial Water Sources in Brazil

**DOI:** 10.1007/s00284-024-03645-5

**Published:** 2024-05-07

**Authors:** Dândrea Driely de Melo Ferrari, Solange Costa Lima, Raquel Lima Figueiredo Teixeira, Marcia Quinhones Pires Lopes, Sidra Ezídio Gonçalves Vaconcellos, Edson Silva Machado, Philip Noel Suffys, Harrison Magdinier Gomes

**Affiliations:** 1 Laboratório de Biologia Molecular Aplicada a Micobactérias, Instituto Oswaldo Cruz (Fiocruz), Rio de Janeiro, RJ 21040-360 Brazil; 2 Conforlab Engenharia Ambiental, São Paulo, SP 04612-002 Brazil

## Abstract

*Legionella pneumophila* (Lp) is a Gram-negative bacterium found in natural and artificial aquatic environments and inhalation of contaminated aerosols can cause severe pneumonia known as Legionnaires’ Disease (LD). In Brazil there is hardly any information about this pathogen, so we studied the genetic variation of forty *Legionella* spp. isolates obtained from hotels, malls, laboratories, retail centers, and companies after culturing in BCYE medium. These isolates were collected from various sources in nine Brazilian states. Molecular identification of the samples was carried out using Sequence-Based Typing (SBT), which consists of sequencing and analysis of seven genes (*flaA*, *pilE*, *asd*, *mip*, *mompS*, *proA,* and *neuA*) to define a Sequence Type (ST). Eleven STs were identified among 34/40 isolates, of which eight have been previously described (ST1, ST80, ST152, ST242, ST664, ST1185, ST1464, ST1642) and three were new STs (ST2960, ST2962, and ST2963), the former identified in five different cooling towers in the city of São Paulo. The ST1 that is widely distributed in many countries was also the most prevalent in this study. In addition, other STs that we observed have also been associated with legionellosis in other countries, reinforcing the potential of these isolates to cause LD in Brazil. Unfortunately, no human isolates could be characterized until presently, but our observations strongly suggest the need of surveillance implementation system and control measures of *Legionella* spp. in Brazil, including the use of more sensitive genotyping procedures besides ST.

**Supplementary Information:**

The online version contains supplementary material available at 10.1007/s00284-024-03645-5.

## Introduction

*Legionella* sp. is a genus composed of aerobic, Gram-negative, and flagellated bacteria, which includes 59 species, three subspecies and more than 70 serogroups, mostly isolated from natural and artificial aquatic environments [1]. Although all species of the genus have pathogenic potential, the specie *L. pneumophila* (Lp) is responsible for 90% of the cases of legionellosis around the world [2]. These bacteria can cause Legionnaires’ Disease (LD), an atypical and sometimes fatal pneumonia, or Pontiac Fever, a mild self-limiting flulike illness. Both clinical forms occur through inhalation of contaminated aerosols [3].

Although *Legionella* are present in natural environments, outbreaks of legionellosis are mainly associated with artificial environments, due to their favorable conditions for colonization such as temperatures between 25 °C and 42 °C, stagnation zones, organic contamination and the presence of protozoa, natural hosts of *Legionella* [4]. Furthermore, production of aerosols by artificial water systems such as cooling towers, spas, and hot spring baths create conditions for access to the human respiratory system, having turned LD into an emerging disease since the seventies [1].

Consequently, surveillance of such artificial aquatic systems for the presence of *Legionella* is the most important intervention to prevent the disease and the study of these microorganisms allows the understanding of epidemiological factors involved and their pathological potential.

Currently, there are several techniques used for the molecular characterization of Lp, such as Variable Number of Tandem Repeats (VNTR); Pulsed-Field Gel Electrophoresis (PFGE); and Amplified Fragment Length Polymorphism (AFLP). However, the European Working Group for *Legionella* Infections (EWGLI), recently renamed European Study Group for *Legionella* Infections (ESGLI), developed a standardized procedure for the molecular typing of Lp, based on the amplification and sequencing of seven genes (*flaA*, *pilE*, *asd*, *mip*, *mompS*, *proA,* and *neuA*), establishing as such their Sequence-based Type (SBT) [5].

In Brazil, there is hardly information about presence of *Legionella* species and if any, based only on serotyping, a procedure lacking discriminative power and lacking information on genetic variability. Therefore, this study aims to carry out the molecular characterization of Lp isolates from environmental sources from different Brazilian states through the SBT technique.

## Materials and Methods

### Sampling

Forty isolates of *Legionella* spp. were collected during 2015, 2018, and 2019 and kindly provided by Conforlab (São Paulo), a company that provides environmental quality control services. These isolates were obtained by collecting 1 L of water from different sources, locations, cities, and states in Brazil (Table S1). Water samples were collected in hotels (21), mall (1), retail centers (4), laboratories (2), food industry (1), and a cleaning company (1).

### Sample Processing and Isolation of *Legionella* spp.

Identification of *Legionella* spp. in the water samples was performed in accordance with the parameters established by ISO 11731, which consists of direct inoculation or inoculation after filtration. During the first procedure, a 200 μL aliquot of 1 L sample was inoculated directly into the selective culture medium buffered charcoal yeast extract (BCYE) agar (Sigma-Aldrich, India), supplemented with glycine (3 g/L), vancomycin hydrochloride (1 mg/L), polymyxin B sulfate (80,000 UI/L), and cycloheximide (80 mg/L) (GVPC; Sigma-Aldrich, Brazil), and incubated at 35º C in an atmosphere of 2.5% CO_2_ for a period of up to 14 days.

For the second procedure, the rest of the collected material was concentrated by filtration on a sterile cellulose membrane with porosity between 0.2 and 0.45 µM and 47 mm of diameter. This membrane was then transferred to a 50 mL tube containing 10 mL of sterile distilled water, 1 mL of 0.2 M HCl added and incubation at RT for 15 min. To neutralize the solution, 1 mL of 0.1 M KOH was added, and 0.1 mL aliquots were inoculated into Petri dishes containing selective BCYE + GVPC medium. The plates were incubated at 35º C in an atmosphere of 2.5% CO_2_ for up to 14 days.

Confirmation of the presence of members of the genus *Legionella* spp. was achieved by incubating the colonies in BCYE medium without the addition of L-cysteine or in blood agar medium, as species of this genus only grow in the presence of this amino acid.

### DNA Extraction

One or two loops of bacterial mass were transferred to a microtube with 1 mL of sterile water and centrifuged at 15,000×*g* for 10 min. The supernatant was discarded, and the pellet was resuspended in 100 µL of RNase buffer (Resuspension Buffer with RNase A – R4) and 5 µL of lysozyme (50 mg/mL). The samples were incubated for 10 min followed by a new step of incubation with 500 µL of lysis buffer (L14) and 10 µL of proteinase K at 80 °C for one hour. All solutions except lysozyme are components of the ChargeSwitch® gDNA Mini Bacteria Kit (Invitrogen, California, USA).

After cell lysis, bacterial DNA was purified by binding to the magnetic beads (ChargeSwitch® Kit) (Invitrogen, California, USA) according to the manufacturer’s protocol and submitted to the magnetic rack (MagnaRack™ Magnetic Separation Rack) (Invitrogen™ / Thermo Fisher, California, USA). DNA quantification by nanodrop is listed in supplementary Table S2.

### Sequence-Based Typing

*Legionella* spp. DNA was subjected to SBT technique according to the protocol proposed by ESGLI, which consists of seven genes fragments amplification (Table S2), followed by sequencing of PCR products and analysis by Sequence Quality Tool of SBT-database [5, 6]. The reference sequences and primers used for SBT scheme are summarized in Table S3. For each sample seven PCR reactions was done. The reaction mixture and conditions used were 1 U de Taq polimerase, 1X PCR buffer, 2,5 mM MgCl2 (*Taq DNA Polymerase Recombinant*™ kit (Invitrogen™, California, USA); 0.2 mM dNTPs, 0.2 mM of primers forward and reverse, and 4 ng/μL of DNA strain, totaling a volume of 50 µL. Amplification by PCR was performed using the Veriti™ 96-Well Fast Thermal Cycler (Applied Biosystems, California., USA) at the following conditions: denaturation at 95 °C for 5 min followed by 35 cycles of denaturation at 95 °C for 30 s, annealing at 56,5 °C for 30 s, elongation at 72 °C for 35 s, and final elongation at 72 °C for 5 min. PCR products were analyzed by agarose gel electrophoresis. As a positive control DNA from strain *L. pneumophila* ATCC 33152 was used, and PCR products were purified using the *ChargeSwitch™ PCR Clean-Up*™ Kit (Invitrogen). Both strands of the amplicons were sequenced with a model ABI 3730xL sequencer (Applied Biosystems).

Sequence files were submitted and evaluated by the Sequence Quality Tool (SQT) with the help of Dr. Baharak Afshar (European Program for Public Health Microbiology—ECDC). This tool submits the sequences to a second step of control quality and defines the alleles of the seven before mentioned targets for identification of ST. Strains with known allelic profiles were assigned to a corresponding ST in SBT-database, while strains with new alleles or new allelic profiles were assigned to new STs by database curation. Additionally, sequences obtained were also shared on NCBI Sequence Read Archive (SRA) portal and can be accessed using the BioProject code: PRJNA1068997.

### Population Genetics Analysis

After being introduced into an Excel table, the identified STs and the partial allelic profiles were grouped into a dendrogram to verify similarity level between the genotypes. The genetic population structure of the strains was defined by creating a minimum spanning tree (MST) based on allele profiles by using Bionumerics version 7.1 (bioMérieux, Belgium) by creating a similarity matrix using the categorical similarity coefficient. Although this procedure is not the classical sequence analysis MLST based approach with input of complete sequences, we observe that there is an overall ST proximity between isolates based on their alleles.

## Results

### Samples

Forty isolates of *Legionella* sp. (Table S1) were obtained from water samples collected from several sources: faucets (35%), cooling towers (22.5%), showers (20%), boilers (12.5%), a drinking fountain (2.5%), and a water reservoir (2.5%). The source of two other samples (5%) is unclear. Among the 40 samples, seven were isolated in the year 2015, 19 in 2018, and 14 in 2019 (Table S1).

Although isolates were obtained in nine Brazilian states, including São Paulo (SP), Rio de Janeiro (RJ), Minas Gerais (MG), Paraná (PR), Rio Grande do Sul (RS), Sergipe (SE), Pernambuco (PE), Bahia (BA), and Goiás (GO); 50% of samples were from SP (Table 1). About 70% from the Southeast region of country, where SP state is located, followed by the Northeast region (15%), South region (12.5%), and only one isolated from the Center-West region of Brazil (2.5%). We did not receive isolates from Northern Brazil.

**Table 1:** Isolates number according to state and region

State	*n*	Region	%
MG	5	Southeast	70
RJ	3		
SP	20		
SE	3	Northeast	15
PE	2		
BA	1		
PR	4	South	12.5
RS	1		
GO	1	Central West	2.5

### Sequence-Based Typing

**Fig. 1:**
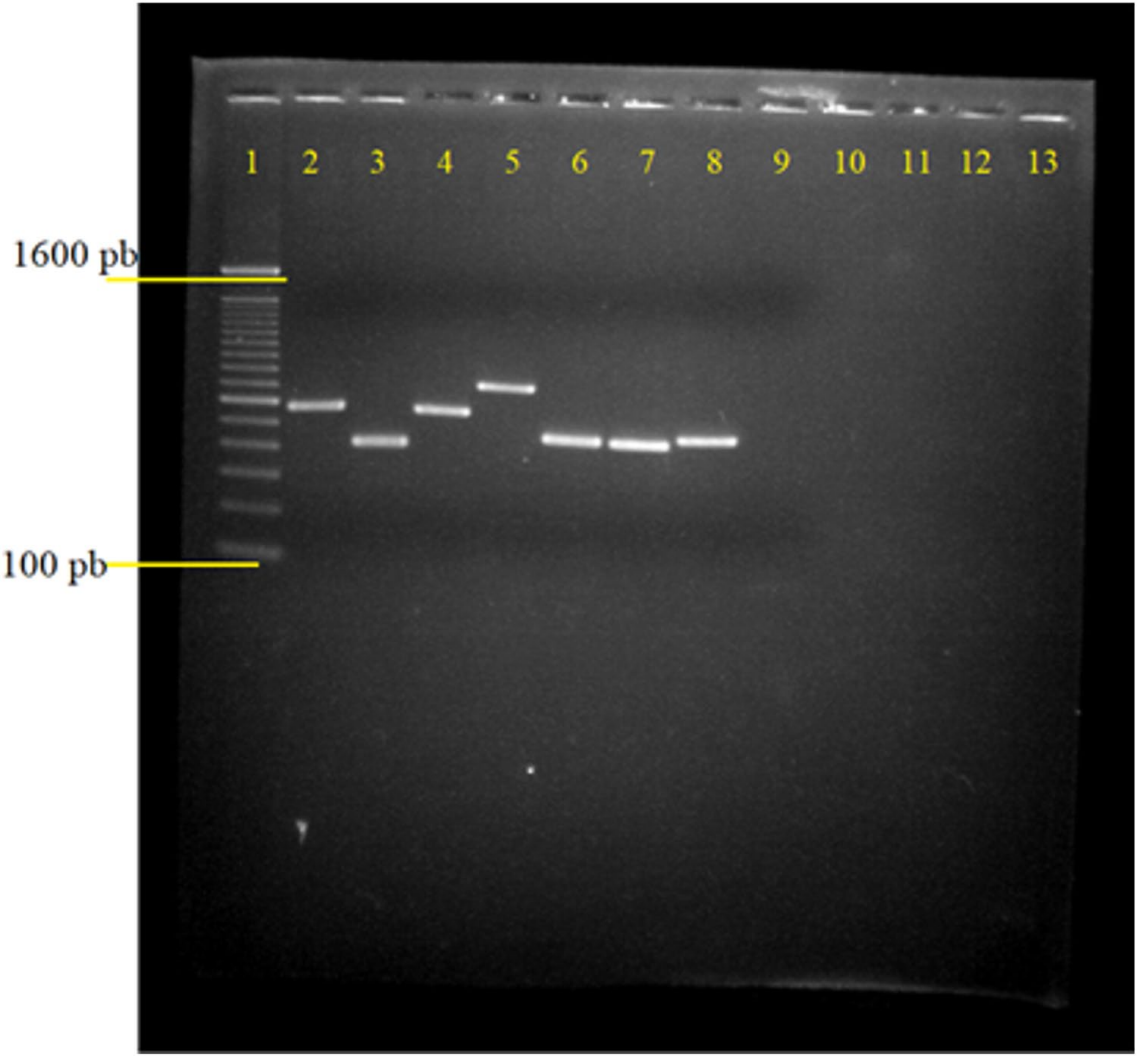
PCR standardization using the *Lp* ATCC 33152 strain for amplification of the seven targets related to the molecular identification of Lp – 1.5% agarose gel in 1X TAE, stained with 0.5 µg/ml ethidium bromide. *1* molecular marker 100 bp ladder; *2* 576 bp fragment of the *asd* gene; *3* 414 bp fragment of the *flaA* gene; *4* 559 bp fragment of the *mip* gene; *5* 711 bp fragment of the *mompS* gene; *6* 459 bp fragment of the *neuA* gene; *7* 460 bp fragment of the *pilE* gene; *8* fragment of 481 bp of the *proA* gene; and *9* negative control

ST is defined from the amplification of seven alleles (Fig. 1) followed by sequencing and analysis by SQT (Figs. S1, S2). It was possible to determine the complete allelic profile for 34 of the 40 isolates and among these, 11 STs were observed (Table 2). Eight had been previously described (ST1, ST80, ST152, ST242, ST664, ST1185, ST1464, and ST1642), while three were new STs (ST2960, ST2962, and ST2963). These three latter did not contain new alleles but were a rearrangement of existing alleles, suggesting the existence of recombination events in the population of *L. pneumophila* isolates.

**Table 2:** Allelic profile and frequency of identified STs

ST	Allelic profile							Sample	
	*flaA*	*pilE*	*asd*	*mip*	*mompS*	*proA*	*neuA*	*n*	%
ST1	1	4	3	1	1	1	1	14	41
ST2960*	1	4	3	1	1	1	21	8	23
ST242	3	10	1	28	1	9	3	2	6
ST664	3	13	1	3	14	9	9	2	6
ST1464	2	10	15	28	19	4	3	2	6
ST80	7	6	3	8	13	11	3	1	3
ST152	1	4	3	1	1	1	3	1	3
ST1185	3	14	16	16	15	13	2	1	3
ST1642	3	6	1	28	14	9	3	1	3
ST2962*	20	26	27	34	46	27	1	1	3
ST2963*	3	6	1	28	14	9	3	1	3

New STs are indicated with a *

Although absence of some markers did not allow us to define the ST of six isolates (due to low sequence quality of some alleles), data of the partial allelic profiles obtained are presented in Table 3. Sequences that define each ST are available in NCBI (BioProject code: PRJNA1068997).

**Table 3:** Isolates with partial allelic profile

Sample ID	Partial allelic profile							Probable sequence type
	*flaA*	*pilE*	*asd*	*mip*	*mompS*	*proA*	*neuA*	
37064	3	4	–	1	14	–	1	Looks like a novel ST
37065	–	12	31	6	48	31	3	Looks like a novel ST
66905	28	21	12	19	0	21	–	No potential STs, likely novel ST
ISHP03	–	0	0	53	14	1	21	No potential STs, likely novel ST
18HP14	3	13	1	3	–	9	1	Consistent with ST or a novel ST
66931	16	21	12	19	31	21	–	Consistent with ST 1317, 1911, 1919, 2247 or new ST

In allelic profile ‘– ‘ means no definition; Looks like a novel ST: the combination of alleles found indicates a new ST; In potential STs: the alleles found do not match with any ST present in the database, indicating that it is a new ST; Consistent with ST or new ST: based on the defined alleles, it is not possible to say whether the isolate belongs to a known or new ST

### Clustered Isolates and Similarity of STs of *Legionella pneumophila*

**Fig. 2:**
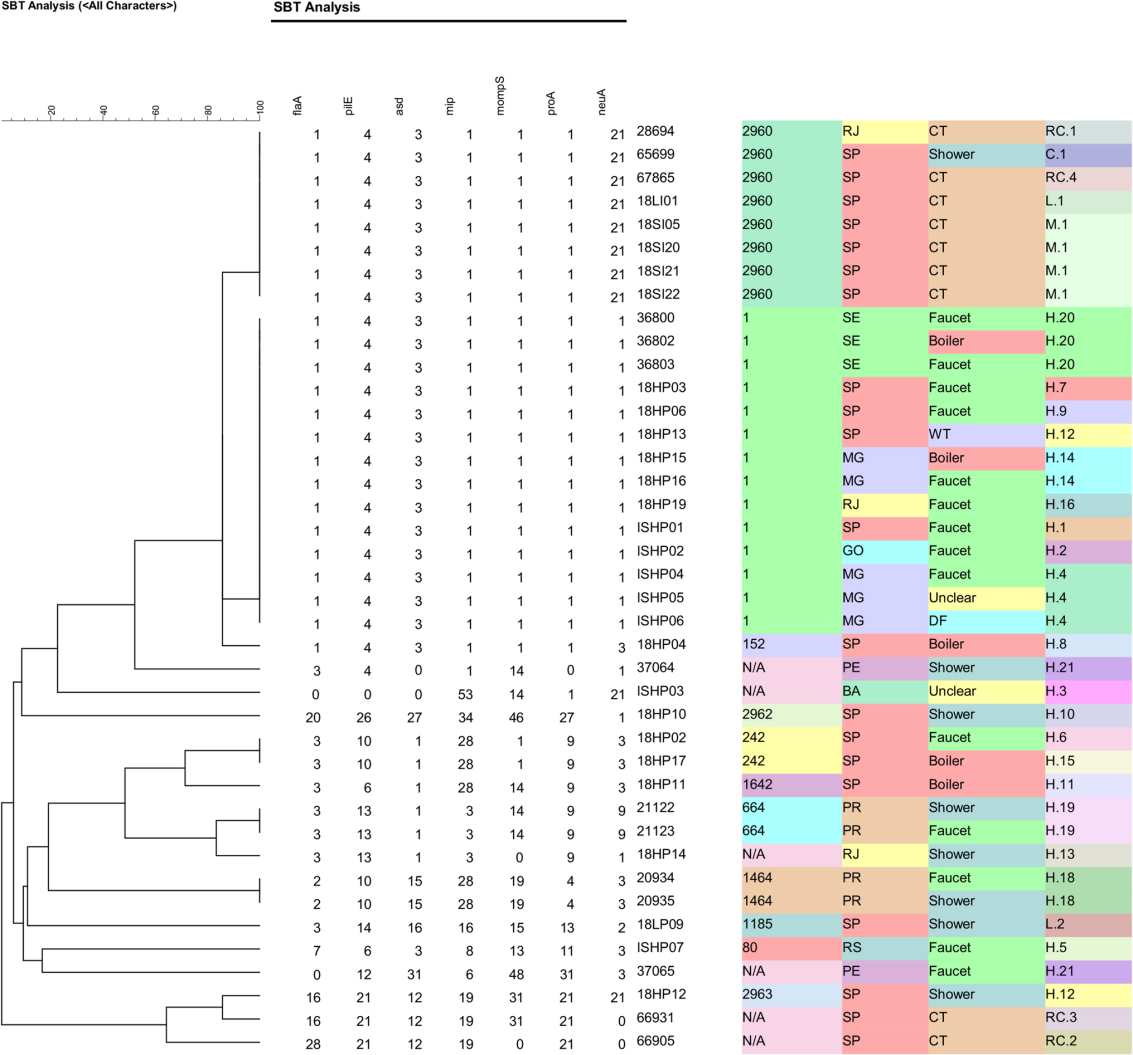
Result of data clustering obtained by the SBT analysis. In the dendrogram, the columns are represented by the seven alleles, isolate ID, ST, state, origin, and local ID. *ST* sequence type, *N/A* ST not defined, *CT* cooling tower, *WT* water tank. *SE* Sergipe, *SP* São Paulo, *MG* Minas Gerais, *RJ* Rio de Janeiro, *GO* Goiás, *RS* Rio Grande do Sul, *PR* Paraná, *PE* Pernambuco, *BA* Bahia. Location ID is defined by a letter + number, so: DF = drinking fountains, H = hotel, M = mall, L = laboratory, RC = retail center, and C = company. Allele code “0” means no allele number definition

Figure 2 represents the UPGMA based comparison of 34 STs (allelic profile based on seven *loci*) and the six partially defined profiles. This figure also summarizes the characteristics of isolates.

Among the 40 isolates, 28 (70%) shared their genotype with at least one other isolate and were therefore clustered. The largest cluster was composed by 14 isolates with ST1, followed by ST2960 composed by eight strains. Three small clusters formed by two isolates each had been isolated and included two isolates from different hotels localized in São Paulo city (ST242); two isolates obtained from different sources of a hotel in Paranaguá city (ST664) and two isolates from different sources of the same hotel in Foz do Iguaçu city (ST1464).

Upon analysis of the differences between genotypes, the two largest clusters (ST1 and ST2960) presented difference in a single locus only (*neuA),* the same being the case for the singleton ST152. Considerable similarity was also observed between ST242, ST1642 and ST664, ST242 and ST1464 presenting only two different loci (*pilE* and *mompS*). Higher variability was observed between ST1642 and ST664, presenting a difference in three *loci* (*pilE*, *mip,* and *neuA*), while ST664 and ST242 share three identical *loci* only, the same occurring with ST242 and ST1464.

Low genotype similarity was observed comparing ST80, ST1185, and ST2962. ST80 shares two alleles with ST152 (*asd* and *neuA*) and shares only the *asd* allele with ST1 and ST2960. ST1185 shares just *flaA* allele with ST242, ST664 e ST1642. Finally, the new ST2962 shares *neuA* allele only with ST1.

When including analysis of incomplete genotypes, the newly described ST2963 presents high similarity with those of incomplete allelic profile of samples 66931 and 66905. Alleles identified in the isolate 66931 are the same as those in ST2963 while among the five defined alleles of sample 66905, four of identical to those defining ST2963.

### STs and Partial Allelic Profile Distribution According to State and Source

Table 4 resumes the distribution of STs according to state of origin and showing that isolates from SP state presenting the largest genetic variability, followed by RJ and PR. Among three samples from RJ, two identified STs showing high similarity (ST1 and ST2960); however, the partial profile of 18HP14 isolate indicates a different genotype with considerable similarity to ST664. In Paraná state, the four isolates belong to two totally different STs (ST664 and ST1464). All samples from MG and the only GO strain belong to ST1, while ST80 was identified only in RS sample. Samples 37064 and 37065 were obtained, respectively, from a shower and a sink of the same hotel in Recife (Table S1), but the alleles identified are not the same in these two strains (37064 = 3, 4, 0, 1, 14, 0, 1) and (37065 = 0, 12, 31, 6, 48, 31, 3).

**Table 4:** Distribution of genotypes (ST) according to origin of the isolates

ST	Allelic profile	*n*	State	Source
ST1	1,4,3,1,1,1,1	14	SE (3), SP (4), MG (5), RJ (1), GO (1)	Faucet, boiler, water tank, drinking fountain
ST80	7,6,3,8,13,11,3	1	RS	Faucet
ST152	1,4,3,1,1,1,3	1	SP	Boiler
ST242	3,10,1,28,1,9,3	2	SP	Faucet and boiler
ST664	3,13,1,3,14,9,9	2	PR	Shower and faucet
ST1185	3,14,16,16,15,13,2	1	SP	Shower
ST1464	2,10,15,28,19,4,3	2	PR	Faucet and shower
ST1642	3,6,1,28,14,9,3	1	SP	Boiler
ST2960	1,4,3,1,1,1,21	8	RJ (1) e SP (7)	Cooling tower and shower
ST2962	20,26,27,34,46,27,1	1	SP	Shower
ST2963	16,21,12,19,31,21,21	1	SP	Shower
na	3,4,0,1,14,0,1	1	PE	Shower
na	0,12,31,6,48,31,3	1	PE	Faucet
na	28,21,12,19,0,21,0	1	SP	Cooling tower
na	0,0,0,53,14,1,21	1	BA	Unclear
na	3,13,1,3,0,9,1	1	RJ	Shower
na	16,21,12,19,31,21,0	1	SP	Cooling tower

NA: not applicable because the STs are undefined

**Fig. 3:**
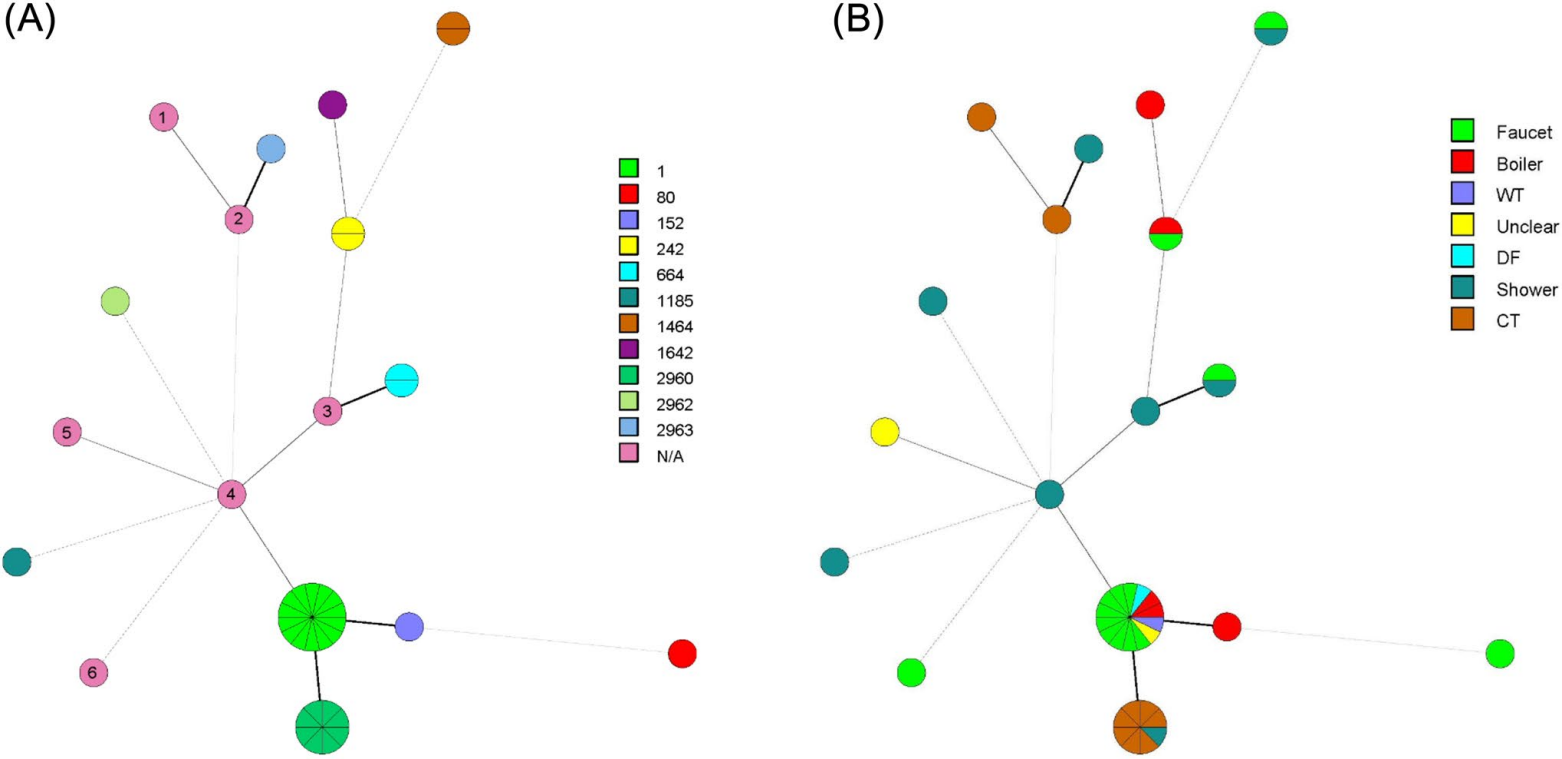
Minimum Spanning Tree based on the STs found (a) and based on the isolates sources (b). STs are represented by circles whose size indicates the number of isolates with this ST. Solid and thick lines connect two STs that differ within a single locus, solid and thin lines connect dual-locus variants, thick dotted lines connect triple-locus variants, and thin dotted lines connect STs that differ at four loci. **A** N/A = ST not defined, only partial allelic profile; numbers 1 to 6 in the circles refer, respectively, to isolates 66905, 66931, 18HP14, 37064, ISHP03, and 37065. **B** *WT* water tank; *DF* Drinking fountain; *CT* cooling tower

Figure 3 demonstrates the general genotypic distribution and the source dependent genotypic distribution of our sampling. Among 14 isolates obtained from tap water, five STs were identified (ST1, ST80, ST242, ST664, and ST1464) in addition to two partial allelic profiles. For five strains from boilers, four STs were found (ST1, ST152, ST242, and ST1642). Six STs were identified for eight isolates from shower samples (ST664, ST1185, ST1464, ST2960, 2962 and ST2963), in addition to two other partial profiles, showing considerable genetic variability in these sample sources (Fig. 2B). For nine isolates from the cooling towers, seven isolates belong to ST2960 and two have a partial allelic profile like the newly described ST2963 (Fig. 2B). Except for cooling towers, no correlation between source and genotype has been established.

## Discussion

Most of the isolates in the present study were derived from hotels, establishments that are known to solicitate quality of their water facilities more frequently. According to CDC-USA [7], frequent visits in hotels can be a risk factor for legionellosis due to exposure of individuals to possible sources of contamination such as showers, taps, bathtubs, and heaters. Furthermore, it is known that the degree of contamination by *Legionella* is higher in water systems of large buildings, probably due to the extension of their hydraulic systems [8].

Among the genotypes obtained presently, ST1 is the largest cluster, and composed by samples of five different states collected in 2015, 2018, and 2019. This result seems consistent with data available in literature, where ST1 is a dominant genotype and widely distributed worldwide well adapted to survival in artificial water sources. In addition, organisms with this genotype are considered the main causative agent of LD globally, supporting its high pathogenicity [9–11].

The second largest cluster is formed by eight isolates with the new ST2960. One sample was isolated from a shower while seven more isolates were from cooling towers in four different locations: a retail center in RJ and one in SP, and a laboratory and a shopping mall, both in SP. Cooling towers use water as to lower temperature during industrial processes or in buildings with a central air conditioning system. This created humid and hot (30–45 °C) environments with stagnation zones, favoring biofilm development. About 21% of water samples collected from cooling towers in Brazilian companies are contaminated with *Legionella* spp. (Conforlab, personal communication); such incidence seems to exist in other countries, including in the USA [12].

Because our study was done on a convenience sampling, the fact that 70% of samples share their genotype with one or more isolates calls attention and could be partly explained by the predominance of samples from São Paulo. Additionally, the collection of more than one water sample from the same place, such as hotels and malls, but from different sources such as showers and faucets, could have contributed to the formation of smaller cluster (ST242, ST664, ST1464).

Since this is the first study of *Legionella* genotyping in Brazil, we included our data of the partial allelic profiles obtained (Table 3) and their participation in the generation of potential new genotypes, adding to genotypic variability. Failure of amplification of some alleles could be due to presence of mutations in primer(s) annealing site or presence of species different from *L. pneumophila*.

As observed with strains 37063 and 37064, isolates obtained from the same place may present different STs. As shown by Sharaby et al. [13], samples obtained by collecting water from the same distribution system may have different genotypes and, consequently, different characteristics. The diversity of alleles present in a given population is what results in genetic variability of species and can promote differences in genes functionality involved, [14]. Genes encoding flagellum-related proteins (*flaA*), pilin (*pilE*), outer membrane protein (*mompS*), macrophage infectivity enhancer (*mip*), and zinc metalloproteinase (*proA*) can interact with external environments; therefore, the adaptation of bacteria to a given environmental source may result in specific and suitable STs for each source, as may have occurred with cooling towers in this study [9]. Furthermore, the findings of this study demonstrate that identical environmental strains can be found at different sampling sites suggest the existence of a complex environmental network that needs further investigation.

Upon comparing our data with those described in literature, eight STs identified presently have been previously described in other countries and some have clearly been associated with Legionnaires’ Disease (Table 5). Isolates with ST1464 have been observed in environmental sources in Indonesia, China, and India, but are apparently not associated with clinical cases in these countries [9]. However, ST664 was associated with two cases of LD in Belgium; one case of unknown origin and another case of travel-associated pneumonia [15].

**Table 5:** The STs associated with clinical and environmental isolates in another countries

ST	Countries with the same SBT	References
1	Worldwide (C and E)	Beatson and Bartley [10], Sreenath et al. [9], and Wüthrich et al. [11]
664	Belgium (C)	Vekens et al. [15]
1464	Indonesia (E), China(E) and India (E)	Sreenath et al. [9]
242	China (E), Japan (E), GBR (C), USA (C)	Guo et al. [16], Zeng et al. [17], Zhan and Zhu [18], and Raphael et al. [19]
80	Sweden (E)	Allestam and Schönning [22]
152	Poland (C and E)	Pancer [20]
1642	Israel (E)	Yakunin et al. [21]
1185	–	Not found in the literature

*C* clinical isolates, *E* environmental isolates. GBR, United Kingdom; USA, United States of America

ST242 genotype was also described in environmental samples from natural and artificial sources in China and in engineered water systems in Japan [16–18]. Interestingly, in Arizona (USA), Japan, and United Kingdom, this allelic profile was observed in clinical samples [16, 19]. ST152 has been observed in water samples from two hospitals in Poland in 2001 and 2005. According to Pancer [20], this ST is frequently seen in strains from hospital environmental sources and in clinical samples from patients with CAP or travel-associated pneumonia.

Isolates with ST1642 have been obtained in hotel water system samples in Israel and are widely distributed in the country just like ST1 [21]. Finally, ST80 was also found in Sweden and is among the most common STs among environmental samples in the country; however, no information is available whether they are associated with clinical cases [22].

It is important to note that although this study exclusively analyzed environmental isolates, the genotypes identified here are also associated with cases of legionellosis in other countries, reinforcing the clinical importance of these samples.

Interestingly, the fact that the new STs (not counting the partial allelic profiles that indicate the probability of other new STs) represent 27% of isolates suggests that a considerable number of so far undescribed Lp genotypes might be present in Brazil´s huge territory. Therefore, recombination events might occur frequently in Lp and this suggests the need to continue monitoring *L. pneumophila* genotypes as recombination events can also affect the adaptability, transmission, and pathogenicity of the isolates.

The major limitation of our study is the relatively small number of isolates that could be genotyped, all being environmental and the majority being from the SP state resulting in a disproportionate distribution among Brazilian states. Nonetheless, this is the first molecular identification study performed with Brazilian isolates and, therefore, may be useful for a timely characteristic’s prediction of isolates in environment before being evidenced in clinical samples.

## Conclusion

Monitoring water quality in distribution systems is essential to verify presence of Lp in environment and is the main way to prevent LD. Several countries have strict surveillance for legionellosis, but is absent in Brazil, where control exist only by quality control of water-cooling system upon solicitation and without further notification to the health system. We here present the genetic characterization of *Legionella* in several water samples in nine Brazilian states and the frequency of STs found, including the presence of genotypes of clinical importance in other countries. Furthermore, a considerable number of isolates presented new STs showing that the genetic variability of Lp is larger that so far described. We also reinforce the need to develop more specific water quality standards for the control and prevention of legionellosis, in addition to the implementation of diagnostic and treatment methodologies in country.

### Supplementary Information

Below is the link to the electronic supplementary material.

Supplementary file1 (DOCX 208 kb)

## Data Availability

Data will made available on request. The data that support the findings of this study are available from the corresponding authors, upon reasonable request.
