# Supplementary material for: Genetic Diversity of Legionella pneumophila Isolates from Artificial Water Sources in Brazil
Source: Curr Microbiol. 2024 May 7;81(6):165. doi: 10.1007/s00284-024-03645-5 (PMC11076386; doi:10.1007/s00284-024-03645-5)
Supplement: Supplementary file 1 — Supplementary file1 (DOCX 208 kb) [file 284_2024_3645_MOESM1_ESM.docx]

**CURRENT MICROBIOLOGY**

**Genetic diversity of *Legionella pneumophila* isolates from artificial water sources in Brazil.**

Dândrea Driely de Melo Ferrari^a*^, Solange Lima^b^, Raquel Lima de Figueiredo Teixeira^a^, Marcia Quinhones Pires Lopes^a^, Sidra Ezídio Gonçalves Vaconcellos^a^, Edson Silva Machado Filho^a^; Philip Noel Suffys^a*^, Harrison Magdinier Gomes^a*^

^a^ Laboratório de Biologia Molecular Aplicada a Micobactérias, Instituto Oswaldo Cruz (Fiocruz), 21040-360, Rio de Janeiro, RJ, Brazil.

^b^ Conforlab Engenharia Ambiental, 04612-002, São Paulo, SP, Brazil.

*Corresponding authors:

E-mail adresses: dan.mferrari@hotmail.com (D. Ferrari), psuffys@gmail.com (P. Suffys), magdinier@gmail.com (H. Gomes).

**SUPPLEMENTARY MATERIAL**

**Table S1.** Information on *Legionella* spp. isolates.

| SAMPLE | YEAR | SOURCE | LOCAL ID | CITY | STATE | LATITUDE, LONGITUDE |
| --- | --- | --- | --- | --- | --- | --- |
| ISHP01 | 2015 | Faucet | Hotel - 1 | Moema | SP | -23.601914413356226,  -46.672703364472305 |
| ISHP02 | 2015 | Faucet | Hotel - 2 | Goiânia | GO | -16.684629417935,  -49.26652248569256 |
| ISHP03 | 2015 | Undefined | Hotel - 3 | Vera Cruz | BA | -12.950554943477107,  -38.61155483075749 |
| ISHP04 | 2015 | Faucet | Hotel - 4 | Belo Horizonte | MG | -19.91887709484401,  -43.92043350974599 |
| ISHP05 | 2015 | Undefined | Hotel - 4 | Belo Horizonte | MG | -19.91887709484401,  -43.92043350974599 |
| ISHP06 | 2015 | Drinking Fountain | Hotel - 4 | Belo Horizonte | MG | -19.91887709484401,  -43.92043350974599 |
| ISHP07 | 2015 | Faucet | Hotel - 5 | Porto Alegre | RS | -30.036272631574175,  -51.1881465796801 |
| 18HP02 | 2018 | Faucet | Hotel - 6 | São Paulo | SP | -23.553720803417754,  -46.617350590950764 |
| 18HP03 | 2018 | Faucet | Hotel - 7 | São Paulo | SP | -23.553720803417754,  -46.617350590950764 |
| 18HP04 | 2018 | Boiler | Hotel - 8 | São Paulo | SP | -23.553720803417754,  -46.617350590950764 |
| 18HP06 | 2018 | Faucet | Hotel - 9 | São Paulo | SP | -23.553720803417754,  -46.617350590950764 |
| 18HP10 | 2018 | Shower | Hotel - 10 | S. Bernardo do Campo | SP | -23.70157956087783,  -46.55580258212169 |
| 18HP11 | 2018 | Boiler | Hotel - 11 | São Paulo | SP | -23.553720803417754,  -46.617350590950764 |
| 18HP12 | 2018 | Shower | Hotel - 12 | S. Bernardo do Campo | SP | -23.70157956087783,  -46.55580258212169 |
| 18HP13 | 2018 | Water Tank | Hotel - 12 | S. Bernardo do Campo | SP | -23.70157956087783,  -46.55580258212169 |
| 18HP14 | 2018 | Shower | Hotel - 13 | Rio de Janeiro | RJ | -22.89861049327173,  -43.20298883453123 |
| 18HP15 | 2018 | Boiler | Hotel - 14 | Belo Horizonte | MG | -19.91887709484401,  -43.92043350974599 |
| 18HP16 | 2018 | Faucet | Hotel - 14 | Belo Horizonte | MG | -19.91887709484401,  -43.92043350974599 |
| 18HP17 | 2018 | Boiler | Hotel - 15 | São Paulo | SP | -23.553720803417754,  -46.617350590950764 |
| 18HP19 | 2018 | Faucet | Hotel - 16 | Rio de Janeiro | RJ | -22.89861049327173,  -43.20298883453123 |
| 18LI01 | 2018 | Cooling tower | Laboratory - 1 | São Paulo | SP | -23.553720803417754,  -46.617350590950764 |
| 18LP09 | 2018 | Shower | Laboratory - 2 | São Paulo | SP | -23.553720803417754,  -46.617350590950764 |
| 18SI05 | 2018 | Cooling tower | Mall - 1 | São Paulo | SP | -23.553720803417754,  -46.617350590950764 |
| 18SI20 | 2018 | Cooling tower | Mall - 1 | São Paulo | SP | -23.553720803417754,  -46.617350590950764 |
| 18SI21 | 2018 | Cooling tower | Mall - 1 | São Paulo | SP | -23.553720803417754,  -46.617350590950764 |
| 18SI22 | 2018 | Cooling tower | Mall - 1 | São Paulo | SP | -23.553720803417754,  -46.617350590950764 |
| 20934 | 2019 | Faucet | Hotel - 18 | Foz do Iguaçu | PR | -25.515710577139977,  -54.57992727778701 |
| 20935 | 2019 | Shower | Hotel - 18 | Foz do Iguaçu | PR | -25.515710577139977,  -54.57992727778701 |
| 21122 | 2019 | Shower | Hotel - 19 | Paranaguá | PR | -25.515106982155086,  -48.51794596240212 |
| 21123 | 2019 | Faucet | Hotel - 19 | Paranaguá | PR | -25.515106982155086,  -48.51794596240212 |
| 28694 | 2019 | Cooling tower | Retail center - 1 | Rio de Janeiro | RJ | -22.89861049327173,  -43.20298883453123 |
| 36800 | 2019 | Faucet | Hotel - 20 | Aracaju | SE | -10.92237354148614,  -37.06609862284662 |
| 36802 | 2019 | Boiler | Hotel - 20 | Aracaju | SE | -10.92237354148614,  -37.06609862284662 |
| 36803 | 2019 | Faucet | Hotel - 20 | Aracaju | SE | -10.92237354148614,  -37.06609862284662 |
| 37064 | 2019 | Shower | Hotel - 21 | Recife | PE | -8.054836290729297,  -34.87793364575171 |
| 37065 | 2019 | Faucet | Hotel - 21 | Recife | PE | -8.054836290729297,  -34.87793364575171 |
| 65699 | 2019 | Shower | Company - 1 | São Paulo | SP | -23.553720803417754,  -46.617350590950764 |
| 66905 | 2019 | Cooling tower | Retail center - 2 | São Paulo | SP | -23.553720803417754,  -46.617350590950764 |
| 66931 | 2019 | Cooling tower | Retail center - 3 | São Paulo | SP | -23.553720803417754,  -46.617350590950764 |
| 67565 | 2019 | Cooling tower | Retail center - 4 | São Paulo | SP | -23.553720803417754,  -46.617350590950764 |

States: SE = Sergipe; SP = São Paulo; MG = Minas Gerais; RJ = Rio de Janeiro; GO = Goiás; RS = Rio Grande do Sul; PR = Paraná; PE = Pernambuco; BA = Bahia.

**Table S2**. Quality and concentration of extracted DNA by Nanodrop.

| Sample ID | ng/𝛍L | 260/280 | 260/230 |
| --- | --- | --- | --- |
| 17861 | 58,9 | 1,75 | 1,17 |
| 20934 | 26,23 | 1,59 | 0,56 |
| 20935 | 164,8 | 1,55 | 0,73 |
| 21122 | 17,44 | 1,53 | 0,76 |
| 21123 | 32 | 1,62 | 0,68 |
| 28694 | 46,7 | 1,8 | 0,34 |
| 36800 | 17,53 | 1,17 | 0,36 |
| 36802 | 169,73 | 1,79 | 1,25 |
| 36803 | 63,96 | 1,92 | 1,22 |
| 37064 | 13,79 | 1,72 | 0,42 |
| 37065 | 331,02 | 1,53 | 0,88 |
| 65699 | 90,46 | 1,82 | 1,14 |
| 66905 | 15,73 | 1,23 | 0,33 |
| 66931 | 60,26 | 1,69 | 0,81 |
| 67865 | 69,96 | 1,68 | 0,82 |
| 18HP02 | 18,03 | 1,44 | 0,76 |
| 18HP03 | 66,31 | 1,75 | 1,29 |
| 18HP04 | 37,87 | 1,67 | 1,07 |
| 18HP06 | 323,18 | 1,64 | 0,89 |
| 18HP10 | 9,99 | 1,02 | 0,45 |
| 18HP11 | 76,16 | 1,75 | 1,06 |
| 18HP12 | 33,31 | 1,52 | 0,88 |
| 18HP13 | 9,1 | 1 | 0,37 |
| 18HP14 | 176,55 | 1,51 | 0,72 |
| 18HP15 | 8,75 | 1 | 0,24 |
| 18HP16 | 9,46 | 1,12 | 0,31 |
| 18HP17 | 31,88 | 1,66 | 0,93 |
| 18HP18 | 1,88 | 0,62 | 0,14 |
| 18HP19 | 51,48 | 1,37 | 0,58 |
| 18LI01 | 141,19 | 1,79 | 1,11 |
| 18LP09 | 44,37 | 1,8 | 1,09 |
| 18SI05 | 52,45 | 1,41 | 0,66 |
| 18SI21 | 1,07 | 0,62 | 0,17 |
| 18SI22 | 4,72 | 0,73 | 0,21 |
| ATCC | 29,25 | 1,83 | 0,99 |
| ISHP01 | 10,12 | 1,67 | 0,51 |
| ISHP02 | 26,15 | 0,94 | 1,67 |
| ISHP03 | 8,21 | 1,07 | 0,52 |
| ISHP04 | 16,07 | 1,19 | 0,45 |
| ISHP05 | 13,96 | 1,56 | 0,49 |
| ISHP06 | 3,18 | 0,71 | 0,18 |
| ISHP07 | 13,09 | 1,39 | 0,56 |

**Table S3.** Reference sequences and primers used for SBT scheme:

| Gene | Genbank reference sequence | Primer name | Position | Primer sequence (5' - 3') | Fragment size (bp) |
| --- | --- | --- | --- | --- | --- |
| *flaA* | X83232 | flaA-587F | 568-587 | GCG TAT TGC TCA AAA TAC TG | 414 |
|  |  | flaA-960R | 981-960 | CCA TTA ATC GTT AAG TTG TAG G |  |
| *pilE* | AF048690 | pilE-35F | dez/35 | CAC AAT CGG ATG GAA CAC AAA CTA | 460 |
|  |  | pilE-453R | 471-453 | GCT GGC GCA CTC GGT ATC T |  |
| *asd* | AF034213 | asd-511F | 487-511 | CCC TAA TTG CTC TAC CAT TCA GAT G | 576 |
|  |  | asd-1039R | 1062-1039 | CGA ATG TTA TCT GCG ACT ATC CAC |  |
| *mip* | AJ496269 | mip-74F | 58-74 | GCT GCA ACC GAT GCC AC | 559 |
|  |  | mip-595R | 616-595 | CAT ATG CAA GAC CTG AGG GAA C |  |
| *mompS* | AF078136 | mompS-450F | 430-450 | TTG ACC ATG AGT GGG ATT GG | 711 |
|  |  | mompS-1116R | 1140-1116 | TGG ATA AAT TAT CCA GCC GGA CTT C |  |
| *proA* | M32884 | proA-1107F | 1090-1107 | GAT CGC CAA TGC AAT TAG | 481 |
|  |  | proA-1553R | 1570-1553 | ACC ATA ACA TCA AAA GCC |  |
| *neuA* | AJ007311 | neuA-196F | 176-196 | CCG TTC AAT ATG GGG CTT CAG | 459 |
|  |  | neuA-634R | 634-611 | CGA TGT CGA TGG ATT CAC TAA TAC |  |

**
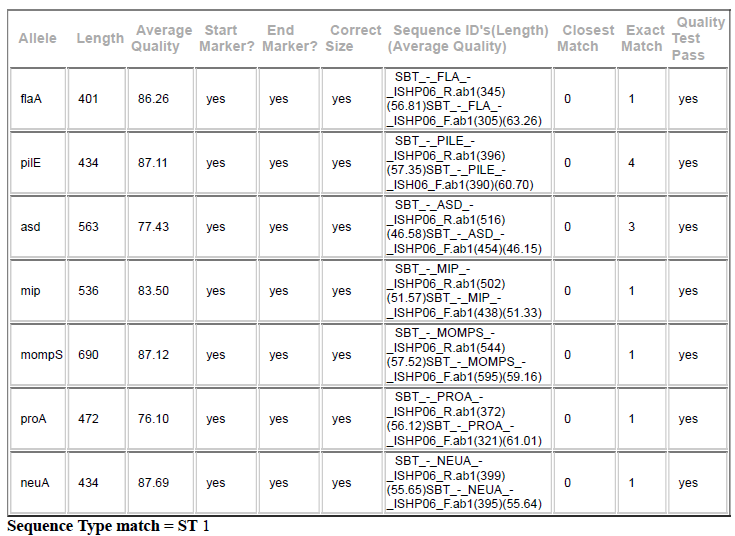
**

**Figure S1.** Example of ST identification by the Quality Assessment tool of the SBT-Database.


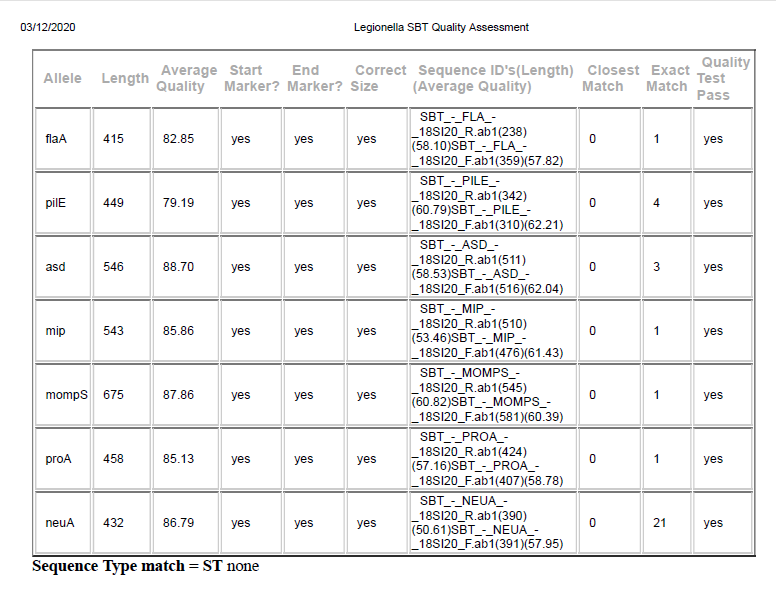


**Figure S2.** Submission of new STs by SBT-Legionella scheme**.** The Allelic profile 1,4,3,1,1,1,21 is new to the database and has been given the Sequence Type 2960.
